# In Silico, Experimental, Mechanistic Model for Extended-Release Felodipine Disposition Exhibiting Complex Absorption and a Highly Variable Food Interaction

**DOI:** 10.1371/journal.pone.0108392

**Published:** 2014-09-30

**Authors:** Sean H. J. Kim, Andre J. Jackson, C. Anthony Hunt

**Affiliations:** 1 Department of Pharmacy and Therapeutics, University of Pittsburgh, Pittsburgh, Pennsylvania, United States of America; 2 Office of Clinical Pharmacology, Food and Drug Administration, Silver Spring, Maryland, United States of America; 3 Department of Bioengineering and Therapeutic Sciences, University of California San Francisco, San Francisco, California, United States of America; University of Catania, Italy

## Abstract

The objective of this study was to develop and explore new, in silico experimental methods for deciphering complex, highly variable absorption and food interaction pharmacokinetics observed for a modified-release drug product. Toward that aim, we constructed an executable software analog of study participants to whom product was administered orally. The analog is an object- and agent-oriented, discrete event system, which consists of grid spaces and event mechanisms that map abstractly to different physiological features and processes. Analog mechanisms were made sufficiently complicated to achieve prespecified similarity criteria. An equation-based gastrointestinal transit model with nonlinear mixed effects analysis provided a standard for comparison. Subject-specific parameterizations enabled each executed analog’s plasma profile to mimic features of the corresponding six individual pairs of subject plasma profiles. All achieved prespecified, quantitative similarity criteria, and outperformed the gastrointestinal transit model estimations. We observed important subject-specific interactions within the simulation and mechanistic differences between the two models. We hypothesize that mechanisms, events, and their causes occurring during simulations had counterparts within the food interaction study: they are working, evolvable, concrete theories of dynamic interactions occurring within individual subjects. The approach presented provides new, experimental strategies for unraveling the mechanistic basis of complex pharmacological interactions and observed variability.

## Introduction

Pharmacokinetic analyses of drug disposition with complex gastrointestinal absorption have been shown to be challenging [1]–[3]. A host of factors including genetic and transcriptional polymorphisms, patient physiology, disease state, experimental or environmental condition, etc. introduce variability and impact pharmacokinetic outcome in a networked, nonlinear, multiscale process, which may confound analysis and hinder reliable prediction. What factors contribute variability, and how do they interconnect to influence disposition? What physiological and pharmacological mechanisms underpin those processes? Answers to these questions are expected to be complex and beyond the grasp of currently available pharmacokinetic methods and modeling tools. Recently we reviewed requirements for modeling and simulation (M&S) approaches [4] that will enable developing deep, exploitable insight into mechanisms responsible for drug disposition (absorption, distribution, metabolism, and excretion) and strategies to enhance predictive and explanatory capabilities of current pharmacokinetic models. The objective of this study is to further explore how synthetic M&S methods as described herein can be applied to better define subject-specific plasma profiles, and provide concrete, parsimonious, and mechanistic explanations in the form of individualized, object-oriented, in silico models.

Previously we introduced prototypal, biomimetic, in silico analogs for gaining insight into subject-by-formulation mechanisms that contribute to intra- and interindividual variability observed in the disposition of an extended-release oral dosage formulation of a Biopharmaceutics Classification System (BCS) Class I drug [5]. By analogs we mean executable software instantiations of plausible generative mechanisms that produce (simulate) behaviors and outcomes that mimic aspects of targeted phenomena (e.g., human drug exposure) [6], [7]. They are grounded on object- and agent-oriented M&S methodologies, which differ from conventional equation-based models and have different yet overlapping uses [4]. We detailed specific steps taken to validate and iteratively refine analogs that corresponded to individual subjects participating in a bioequivalence study. Final validation against dissolution and plasma concentration data required a two-component, heterogeneous gastrointestinal (GI) space, which we hypothesized to map to individualized mechanistic heterogeneity that is a consequence of dosage form–GI tract interactions. A stringent level of similarity was established quantitatively between the simulated and clinical outcomes.

We sought to further elaborate our approach, and engage a scientific M&S process to challenge, falsify, and iteratively evolve the preceding analogs to apply in the more complex case of extended-release felodipine disposition with food interaction [8]. Felodipine is a BCS Class II drug in which in vivo drug dissolution is the rate-limiting step for absorption except at a very high dose [9], [10]. The drug is characterized by variable bioavailability and needs enhancement in dissolution to increase the bioavailability [11], [12]. Given differences between BCS Class I (high solubility and high permeability) and II (low solubility, high permeability) compounds, our expectation was that mechanism changes may be needed to enable the analogs to achieve new validation targets, i.e., generate disposition specific measurements that match the felodipine plasma concentration profiles (hereafter, plasma profiles) as determined by prespecified similarity criteria. To that end we followed an iterative M&S protocol [5], [13], [14] to parsimoniously revise the earlier analog. Several mechanistic variants were explored, subjected to validation, and falsified. Falsification provided specific, useful insight–new knowledge–that guided subsequent analog mechanism revision, and led to discovery of an analog with a new, secondary component connecting to the existing GI module. Parameterizations were found that enabled achieving a predefined level of similarity for all six pairs of referent plasma profiles in fasting and fed conditions.

The parameterized analogs are simple and intuitive yet abstract. Our strong parsimony guideline prevents adding appealing, more realistic detail until it is needed. When executed in simulation, the details of different processes and events occurring within individual analogs can be observed, measured, and can be analyzed in much the same way that real-world counterparts are studied. We hypothesize that all analog processes had counterparts within the food interaction study: they are working, evolvable, concrete theories of dynamic interactions occurring during product dissolution, drug absorption and subsequent disposition within individual subjects. Because the analogs are object-oriented, modular, and intended for reuse and repurposing, it is straightforward to change mechanistic details to simulate additional attributes or experiments, and increase (or decrease) the granularity of analog features. We anticipate that experimenting on such analogs will become an increasingly important research and development strategy for improving formulations, and expanding the “personalized medicine” vision to include complicated, individualizable dosage forms.

## Methods

### Referent studies

Recent publications investigated six healthy volunteers who were administered magnetically labeled extended-release tablets containing felodipine under fasting and fed conditions [8], [15]–[17]. A clinical randomized cross-over study provided dynamically collected tablet’s GI positions and drug release data using magnetic marker monitoring (MMM), a novel imaging technique for the investigation of the behavior of solid oral dosage forms within the GI tract [18]. These data were then modeled using a new mechanism-based approach that simulated tablet movement in the GI tract: the Gastro-Intestinal Transit Time (GITT) model [17]. It is a nonlinear mixed-effects model that relies on some a priori knowledge on tablet transit times inferred from the MMM data. In the study, model characterization was presented as several sequential zero-order drug release rates followed by zero-order transport to the fundus, antrum, and posterior intestinal regions, with first-order absorption across the GI tract. The results indicated two subpopulations characterized by no return to fundus or having one or two returns to fundus.

### Object- and agent-oriented, discrete event M&S approach

Object-oriented and discrete event methods are not new but relatively recent in pharmacokinetic modeling. The methods are used extensively in addressing a variety of engineering and social science problems [19]–[21]. Within the biomedical domain, object- and agent-oriented models have been used for studying systems composed of interacting components exhibiting autonomous, complex, emergent behaviors that are not amenable to closed-form analysis [22], [23]. Object-oriented programming (OOP) enables building software as a set of discrete, interacting, encapsulated units (“objects”) of programming logic [24]. In OOP, objects have state and behavior, much like their real-world (or hypothesized) object counterparts. Object state is maintained using object variables, and executable methods (functions, procedures, and algorithms) define object behavior. For example, drug metabolizing enzymes may be represented as objects having a state (free or substrate-bound) and methods that implement key metabolic events, e.g., substrate binding, oxidization, and product release. Similarly, drug substrates can be represented as mobile objects, each with an internal variable indicating its metabolic state (unmodified, oxidized, reduced, etc.) and methods simulating Brownian motion and other passive movement. Additional variables can be added that specify more fine-grained details like enzyme class, isoforms, and one or more physicochemical properties. Enzyme-substrate interactions occur when a substrate object encounters an enzyme, which may stochastically bind, metabolize, and release product. Prototypal examples are presented in [25], [26]. Agent-based models are executable software devices implemented using OOP techniques. An agent is a quasi-autonomous object that can schedule its own actions within simulation, and adapt to internal and external changes. Rules define the agent’s actions; a more advanced, autonomous agent can set or alter its own rules. Agents typically represent entities–cells, organ systems, organisms, etc.–that exhibit some level of autonomy and ability to engage and interact with the environment. In simulation, an agent senses and is part of its environment, and can choose dynamically with which other agents or objects to interact, when to engage other agents, and which of various actions to take. Importantly, an agent is identifiable by an observer as a cause of an effect. Discrete event M&S methods allow execution of an object-oriented system as a discrete sequence of events in time where each event marks a change of state in the system [27]. A more detailed explanation is provided in [6], [7], [23]. Current limitations of the object- and agent-oriented M&S approach are discussed in [20], [28], [29].

### Framework and in silico analog design

**Figure 1:**
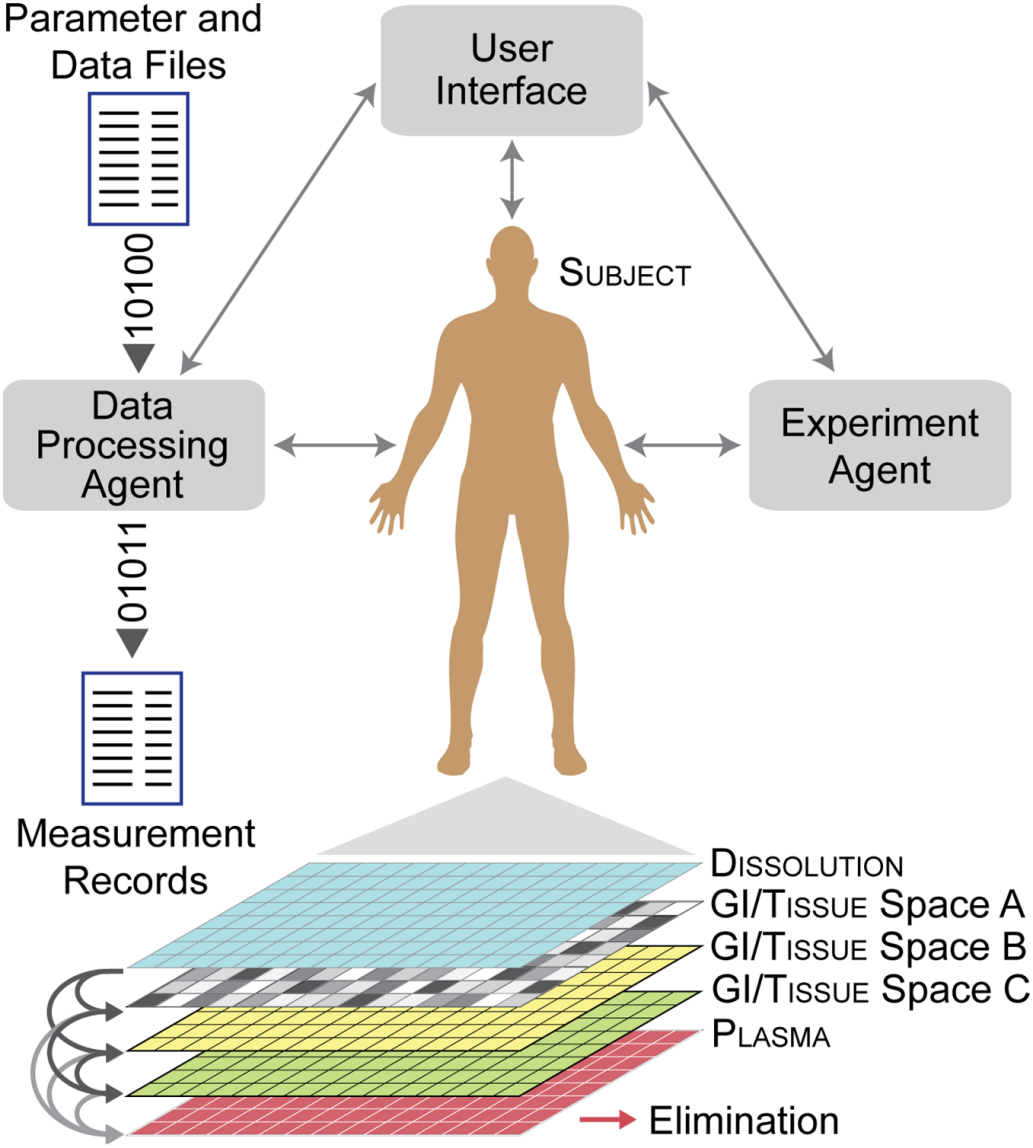
Simulation platform. The computational framework is a coarse grain analog of whole-body drug disposition experiments. It has features to manage, support, and semi-automate simulation and analysis. An object-oriented, agent-based, discrete event model is integrated within the framework; it is an executable software analog of a subject undergoing study. We used two-dimensional, square grids to represent basic physiological features such as plasma and GI tract, which are interconnected to simulate drug movement across individual sites and elimination from the system.

We adapted the in silico, whole body, drug disposition M&S framework from a previous study [5]. The framework illustrated in Fig. 1 was built using an open source, multi-agent simulation software library MASON [30], which supports discrete event, discrete time simulation [27] as well as continuous time modeling. As a pharmacological modeling framework, it has basic components and features for in silico experimentation and analysis, including agents that conduct and manage experiments, process data, and support graphical user interaction, which is available as a stand-alone software package much like NONMEM (ICON plc, Dublin, Ireland) and ADAPT (Biomedical Simulations Resource, Los Angeles, CA) modeling platforms. At the framework’s core is a pharmacologically responsive, in silico analog of a human subject undergoing experiment (Fig. 1). It comprises a set of interconnected two-dimensional grids, components, and event mechanisms that map to a conflation of different physiological features and processes. Component algorithms–programmatic logic statements–govern analog drug (simply drug hereafter) movement between and within individual grid spaces, and eventual elimination from the system. The resulting model differs from closed-form pharmacokinetic models that employ sets of mathematical equations implemented as procedural functions or subroutines (e.g., PREDPP ADVAN in NONMEM). In lieu of equations, the analog comprises objects and algorithms that when executed give rise to measurable dynamic system behavior–individual and aggregate object states that evolve over a sequences of discrete time points and/or continuous time. Time-lapse state changes of every object and event sequences can be measured and recorded for visualization and post-simulation analysis, e.g., drug amount in the analog’s grid sites and objects, the number (and frequency) of metabolic events, the amount excreted unchanged, etc.

**Figure 2:**
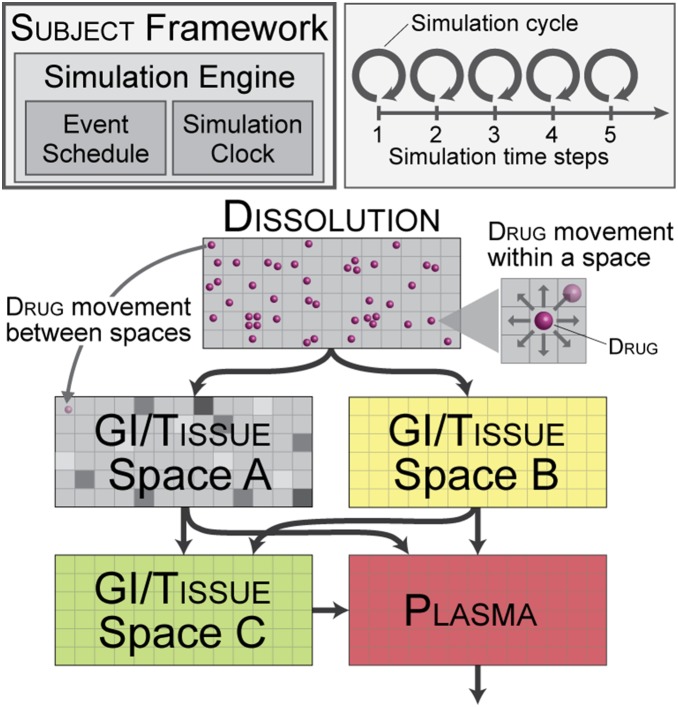
In silico model. The current, validated analog comprises five spaces that map abstractly to the dissolution site, plasma, and GI/tissue features that are hypothesized to impact felodipine disposition. The arrows indicate drug object movement. Drug objects move between interconnected spaces and exit from the plasma grid. Simulated mixing and distribution occurs within each space, which we implemented using a discrete dispersion algorithm. Structural and functional heterogeneity can be introduced at any scale. Spaces shaded differently within a grid (e.g., GI/tissue space A) indicate that their properties can be customized, should that be needed to represent heterogeneity. Relative to our prior work [5], GI/tissue spaces A and B map to the GI tract and interaction space, respectively. The in silico model is a discrete event system, i.e., system dynamics emerge as a consequence of events executing at discrete time points. Drug movements within and between spaces are represented as events and scheduled for every simulation cycle. Scheduling of events and simulation time management are handled automatically by a simulation engine (MASON library) instantiated within the framework, which includes an event scheduler and simulation clock.

In our earlier study, simple, in silico mechanisms consisting of four structures–dissolution, GI, interaction space, and plasma–sufficed for generating varied, multi-peak plasma concentration outcomes that matched individual plasma profiles of drug X from a bioequivalence study [5]. Felodipine disposition characteristics are different, but the basic processes and general, subject physiology are assumed to be similar between the two studies, which allowed us to begin by adopting the earlier analog. We then followed the iterative refinement protocol to extend the analog phenotype and achieve the new set of validation targets without having to reengineer the whole system, and without compromising already validated features and behaviors [13], [14]. The protocol starts with specifying referent attributes to be targeted, e.g., drug and metabolite concentration-time data, histological and biochemical measurements, morphological characteristics, etc. Next, an initial (small) subset of attributes is selected, and an analog is constructed, tested, and revised iteratively until the analog exhibits the targeted attributes within a prespecified level of similarity, thereby achieving a level of validation. Once the iteration completes, the process then repeats with the addition of one or more attributes from the curated list and/or an increase in the level of similarity threshold for validation. A goal of iterative refinement is to shrink plausible mechanism space [7]. For felodipine disposition modeling, several iterations were undertaken in which we challenged analog mechanisms, falsified several and revised them to improve outcome similarity to referent plasma profiles. Shown in Fig. 2 is the analog that achieved validation. It has an additional grid space (GI/tissue space C), which connects from existing GI/tissue spaces to plasma. Other analog features were unchanged: Dissolution connects to GI/tissue spaces A and B, which individually connect to plasma; no interconnection between them was needed. Relative to our earlier work, GI/tissue spaces A and B map to GI tract and interaction space, respectively.

### Analog parameters

Table 1 lists parameters that are accessible to the user for analog configuration, initialization, and simulation execution. The parameters define structural component specifications (e.g., grid sizes) and constraints that apply to drug disposition such as the probabilities and extent of drug movement between and within individual grids. They remain constant during execution unless changed externally by the user or the Experiment Manager. Table 1 parameters are not part of the observations made on the analog: it is the objects’ internal states (e.g., drug amount in plasma) that are measured and correspond to single point variables in equation-based models. They also do not correspond to derived pharmacokinetic parameters like clearance, half-life, and volume of distribution. As described below, pharmacokinetic parameters are determined from measurements (e.g., drug plasma concentrations) made on the in silico analog while it executes, which simulates a human subject undergoing study.

**Table 1:** In silico analog parameters.

Parameter(s)	Default Value	Description
*GridWidth, GridHeight*	100	Grid width (x-axis) and height (y-axis) applied to all grids
*XScale*	1	Scalar factor applied to map simulation cycles to real time
*YScale*	120	Scalar factor applied to the dose fraction in plasma to account for differences between dissolution and plasma concentration measures
*InitDose*	10000	Total drug amount (number of drug objects) initialized and distributed within the dissolution grid at the start of simulation
*DtoGDelay*	1	Initial delay (number of time steps) before initiating drug transfer from dissolution to GI/tissue spaces A and B
*DtoGFract*	0.1	Fraction of drug amount transferred from individual dissolution sites to corresponding GI sites. Valid range: 0–1 inclusive
*DtoGProb*	0.8	Probability of transfer from individual dissolution sites to GI/tissues spaces A and B. At the start of a transfer event, a pseudo-random number, 0≤*p*≤1, is generated. Transfer occurs if *p*≤*DtoGProb*. Valid range: 0–1 inclusive
*DiffGRatio*	1	% of drug transferred to GI/tissue space A. For example, when set to 0.8, 80% of drug transfers to space A, and 20% to space B
*GtoCDelay, GtoCFract, GtoCProb*	0, 0, 0	Initial delay, fraction transferred, and the probability of transfer from GI/tissue spaces A and B to corresponding sites in space C
*GAtoPDelay, GAtoPFract, GAtoPProb, GBtoPDelay, GBtoPFract, GBtoPProb, GCtoPDelay, GCtoPFract, GCtoPProb*	0, 0.1, 0.8	Initial delay, fraction transferred, and the probability of transfer from individual GI/tissue sites to corresponding plasma sites
*PtoEDelay, PtoEFract, PtoEProb*	0, 0.1, 0.8	Initial delay, fraction eliminated, and the probability of elimination from individual plasma sites
*DisperseRate*	0.1	Simulated dispersion rate, which abstractly corresponds to the diffusivity of the target drug. Valid range: 0–1 inclusive
*EvapRate*	0	Loss rate, which specifies the fractional concentration amount evaporated (i.e., dissipated) per dispersion step. Valid range: 0–1 inclusive
*DisperseCount*	2	Number of dispersion step iterations executed per simulation cycle

Default values shown in Table 1 apply automatically when the analog first initializes, which produce a simple, one-peak plasma profile. The parameters can be modified before or during simulation. Changes are delayed and take effect in subsequent simulation runs for the following parameters: *GridWidth*, *GridHeight*, *InitDose*, *MaxCencentration*, *DispersionOn*, and *DisperseCount*. Changes in all other parameters take effect immediately and influence simulation outcome as it unfolds. Individual parameters affect outcome differently, and the effects of one or more parameter changes may be offset by changes in other parameters. Parameters pertaining to drug movement, as described below, can be altered to effect slow or more rapid accumulation of drug in specific spaces. For instance, the parameters (*GAtoPFract*, *GAtoPProb*, *GBtoPFract*, etc.) governing movement from GI/tissue spaces to plasma may be assigned higher values to raise plasma drug concentrations, while increasing the values of *PtoEFract* and/or *PtoEProb* tends to produce a sharper descent and lower plasma concentrations. Delay parameters (e.g., *DtoGDelay*) can be altered likewise to introduce a lag in drug movement between respective spaces and postpone the appearance of drug in plasma. Atypical plasma profiles that exhibit two or more peaks can be obtained by varying *DiffGRatio* and related parameters like *GtoCFract*, *GBtoPProb*, and *GCtoPFract* that control movement between GI/tissue spaces A, B, and C and subsequent transfer to plasma. See Figures S1, S2, S3, S4, S5, S6, S7, S8, S9, and S10 in File S1 for specific examples of how parameter changes impact outcome.

### Drug movement

There are two forms of analog drug movement: inter- and intra-grid, as detailed in [5]. Briefly, movement between interconnected grids occurs with parameter-controlled probabilities. Within each time step, a fraction of drug present is transferred from one grid location to another with some probability. For example, drug movement from GI/tissue space A to plasma is governed by the parameters *GAtoPProb* and *GAtoPFract* that specify, respectively, the probability of movement occurring and the fraction of drug transferred at grid location (*x*, *y*) in each simulation cycle. These parameters impact the rate of drug movement between interconnected spaces: lower probability and/or fraction values lead to a reduction in the mean rate of drug movement. Intra-grid movement effects distribution within (but not between) plasma, GI, and other structures. It uses a discrete approximation algorithm for local dispersion, which executes independently of inter-grid movement. The parameter *DisperseCount* specifies the number of algorithm iterations executed per simulation cycle; higher number reflects more rapid distribution. Within a simulation cycle, grid-to-grid movement events execute in the following sequence: 1) elimination from plasma; 2) movement from GI/tissue spaces to plasma; and 3) movement from dissolution to GI/tissue spaces. All other events execute in pseudorandom order.

### System dynamics

Simulation time advances in discrete time steps 0, 1, 2, …, *t*. One simulation cycle is executed to completion per time step. Algorithms implementing drug movement repeat some number of times within a simulation cycle. By default, grid-to-grid movement at each grid site (*x*, *y*) is computed once per cycle following the sequence describe above. For intra-grid movement, the *DisperseCount* parameter (Table 1) sets the number of iterations that the algorithm executes; the default number is two, i.e., the movement occurs twice within a simulation cycle. Algorithm executions (events) are scheduled and managed automatically by MASON’s simulation controller. All events are discrete: each event is assumed to occur at a singular instance of time. System dynamics emerge as a consequence of discrete events executed in a sequence that change the system state. System state (e.g., the drug amount in plasma and how it changes over time) is what we observe and measure.

### Comparison of felodipine plasma concentrations

We defined similarity criteria to determine whether a simulated outcome is sufficiently similar to features of the referent plasma profile. Analogs achieving the criteria are considered valid (until falsified). Recent examples are provided in [25], [26]. The similarity criteria define upper and lower bounds around the referent values, and require that a specified number or ratio of simulated values occur within those bounds. Narrower bounds are more stringent and harder to achieve, much like requiring regression models (e.g., NONMEM) obtain lower objective function values. For this study, validation required that all nonzero values lie within a band +/−30% of referent values, and additionally, four or more values lie within +/−10% of referent values. The stringency level was specified so that analog plasma profiles achieving the criteria match their referent more closely than did those obtained using the nonlinear mixed-effects approach [17].

The units, dimensions, and/or objects to which a variable or model constituent refers establish groundings. Our analogs have been designed to use relational grounding for maximum flexibility, where variables, parameters, and input/output (I/O) are in units defined by other system components. That contrasts with absolute grounding, in which variables, parameters, and I/O are grounded to real-world units like µm, kg, ml, etc. Differences along with discussions of when to prefer one, the other, or some hybrid are discussed in [4], [31]. Because the analogs are relationally grounded, we need separate mapping to translate simulation metrics to real-world units. Direct comparison of time-plasma concentration data required us to map both time and plasma concentrations. For mapping time, we applied the following: *t_r_* = (*t_s_* – *offset*) * *XScale*, where *t_r_* is referent time (h), *t_s_* is simulation time step, and *offset* = 1. The *offset* factor accounts for the simulation start time, which can be set to any discrete number, and the actual execution timing of measurements. In the current study, we set the start time to zero, i.e., the initial simulation cycle executes at *t_s_* = 0, and measurements made at the end of cycle (but prior to starting next cycle), hence the offset of one time step. For plasma drug values, we used: *C* = *A_plasma_* * *d*/*YScale*, where *C* is plasma concentration (nmol l^−1^), *A_plasma_* is the total drug amount in plasma space, and *d* is the referent dose. *XScale* and *YScale* are scaling factors as described in Table 1; further detail is provided in [5].

### Simulation experiment design

The following describes design and execution of simulation experiments. First, the top-level system components–Experiment Manager and Data Processing Agent–were initialized to set up and manage individual simulations. Next, the Experiment Manager generated a new instantiation of the analog intended to mimic a study participant. A pseudo-random number generator was initialized concurrently for use by the algorithms governing drug movement. The analog instantiation was parameterized to values specified by the user or default values if not specified (e.g., during initial run). At the start of simulation, the dissolution grid was initialized to the specified dosage value; GI/tissue grid values were set to zero as baseline concentrations. The simulation started following initialization, and lasted for some number of simulation cycles. In silico drug movement occurred within the analog per simulation cycle as described above. Simulated plasma drug concentrations and other state values were measured every cycle. At simulation’s end, the recorded measurements were written to files managed by the Data Processing Agent, and the analog instantiation was expunged from the system. A new analog instantiation was created for each simulation experiment.

### Pharmacokinetic analysis

To further characterize and compare disposition outcomes, we estimated pharmacokinetic parameters from the referent and analog plasma concentration curves using noncompartmental methods (Phoenix WinNonlin 6.0, PharSight Corp., St. Louis, MO). Directly estimated parameters were peak plasma concentrations (C_max_, nmol l^−1^), peak time (t_max_, h), and lag time (t_lag_, h). Area under the plasma concentration-time curve over all time points (AUC_all_, nmol l^−1 ^h) was calculated using the linear trapezoidal method. No estimation of terminal phase parameters was made with the observation time window <8 h.

### Implementation tools

We implemented the framework using a multi-agent simulation library, MASON [30] and a general-purpose programming language Java [32]. Java is an object-oriented programming language, which differentiates from traditional procedural programming languages like Fortran (NONMEM and ADAPT are Fortran-based). We used R 2.15.1 (http://www.r-project.org) for data analysis and graph production. GetData Graph Digitizer (http://getdata-graph-digitizer.com) was used to digitally capture the referent and simulation data from [17].

## Results

The analog instantiation in Fig. 1 represents a human subject undergoing study. Analog execution simulates an experiment being conducted on that subject. Given a referent plasma profile, a new analog instantiation was initialized, and parameterized with default values (Table 1). Larger grid sizes did not measurably alter outcome (not shown). Once initialized, we executed the model for a set number of cycles, and measured drug levels in plasma, GI, etc. after each cycle. Similarity criteria were applied to determine if validation targets were achieved. If not, we adjusted parameter values and repeated the simulation. Adjustments were based on heuristics gained from examining failures and how changes in individual parameters impact overall disposition. When parameter adjustments failed to achieve our similarity criteria, the analog’s particular mechanism was falsified (along with all identically performing finer grain variants), thereby shrinking the space of plausible coarse grain mechanisms [7]. Achieving the similarity criteria typically required ∼20–30 iterations; complex profile shapes necessitated more iterations. The analog was falsified if we failed to discover a satisfactory match within 100 iterations for any one of the referent plasma profiles.

Starting with the analog from [5], we discovered parameterizations that enabled it to validate against several referent plasma profiles but failed to do so for others, which falsified that analog. We subsequently conducted several cycles of mechanism revision, testing, and falsification to discover the new variant that validated against all six pairs of referent profiles. An early, simple revision maintained the same grid spaces with new connections between the GI/tissue spaces A and B, however we failed to find parameterizations to achieve validation. Next, an additional space was added, and connected in parallel to the existing GI/tissue spaces for increased heterogeneity, but that also failed to validate. Several, subsequent mechanism revisions had a new grid connecting from the dissolution to GI/tissue space A, from the GI/tissue space A to B, or reciprocal links between GI/tissue spaces. Some produced improved outcomes, however all such variants failed validation (results not shown). We continued the process until we arrived at the Fig. 1 analog. It achieved validation targets using an additional GI/tissue space as shown in Fig. 2.

**Figure 3:**
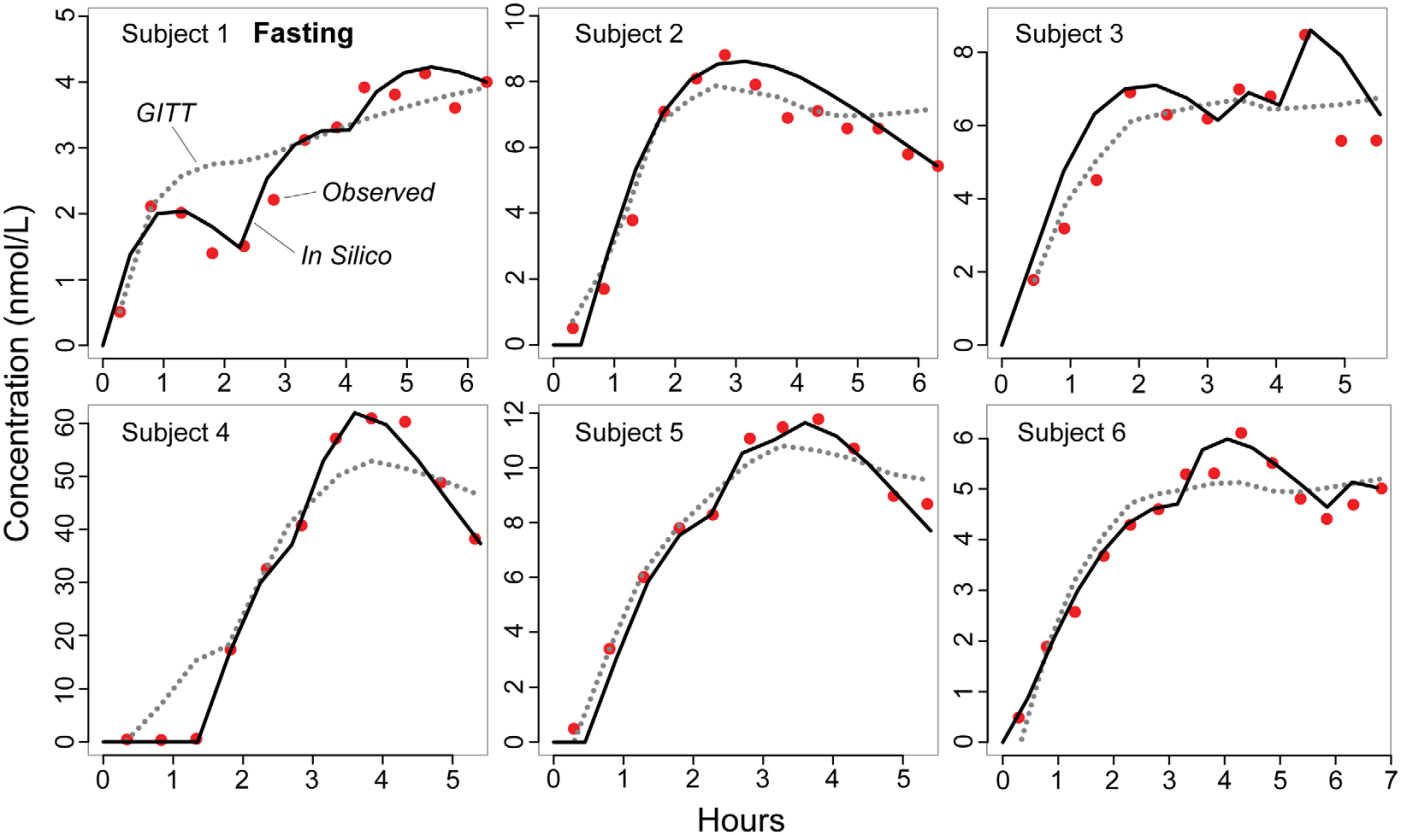
Drug plasma concentration vs. time profiles of individual subjects under fasting conditions. We used the analog from Fig. 2. To simulate a subject undergoing study, we started with default parameter values to initialize the analog and execute simulation. Analog plasma concentration was recorded each time step and compared with the referent profile at the end of simulation. If the outcome failed to validate, i.e., satisfy a prespecified level of similarity, we adjusted parameter values and repeated simulation. We repeated the process until the similarity was achieved. The analog parameterized to Table 2 values produced individual outcomes (black lines) that validated against the corresponding referent profiles. For comparison, the GITT model’s predicted concentrations (dotted curves) are reproduced from [17]. Observed concentrations (red) are from [8].

**Figure 4:**
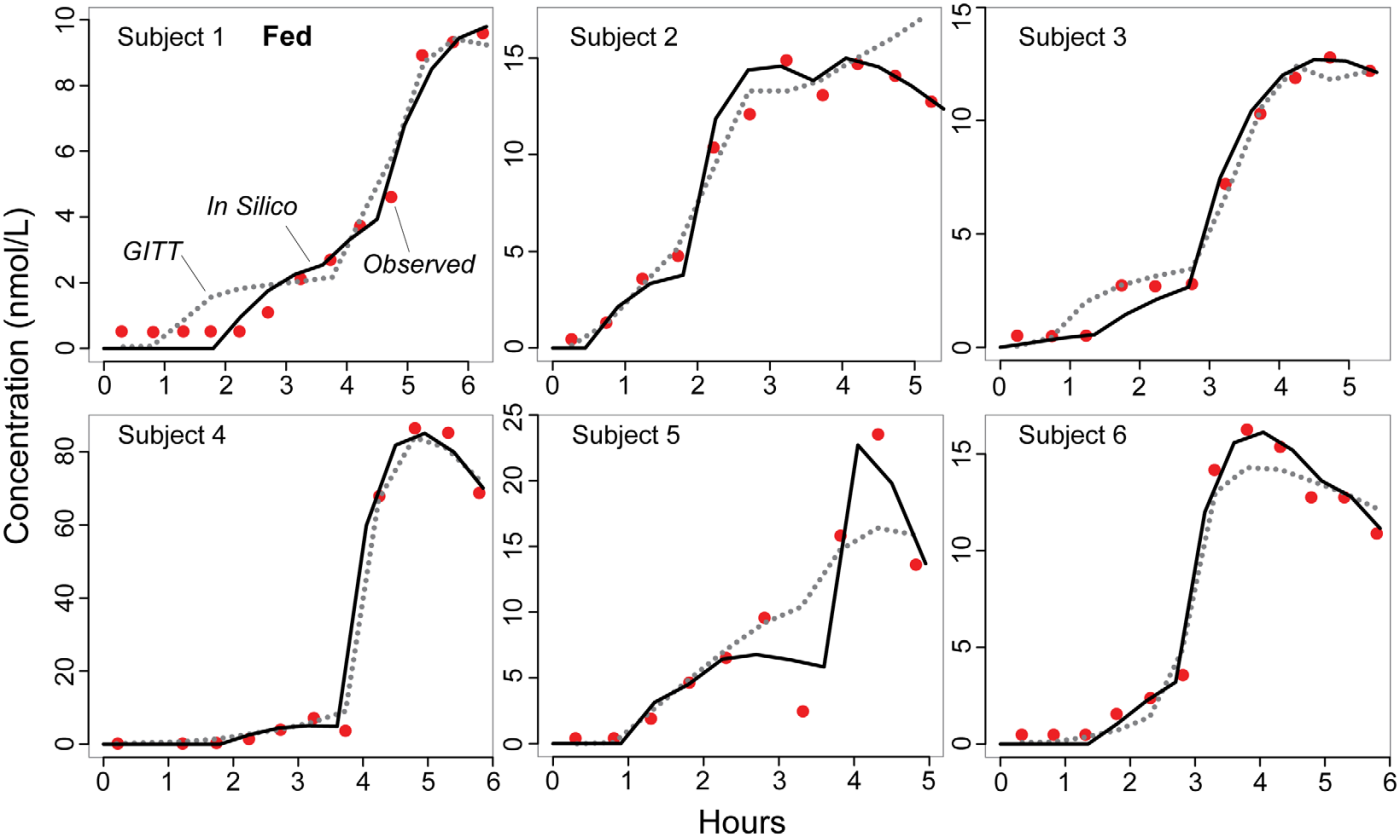
Felodipine plasma concentrations following food intake. We used the same protocol described in Fig. 3 and in Methods to simulate drug disposition under the fed condition. Red circles: observed concentrations [8]; black lines: analog outcomes; dotted curves: the GITT model’s predicted concentrations [17].

Figures 3 and 4 present in silico plasma profiles from the final, validated analog under fasting and fed conditions, respectively, shown with the GITT model predictions from [17]. All achieved the similarity criteria with the analog parameterized to Table 2 values, which we obtained after performing many iterations of parameter adjustment and/or mechanism refinement as described above, and selecting for those that performed better based on visual and quantitative comparisons [5]. Except for analog Subject 2′s profile under the fasting condition, validation required some portion of drug to move through GI/tissue space C to plasma. Also, absorption in most subjects engaged the heterogeneous GI mechanism, with more extended inflow, and delayed outflow from space B, which helped to capture the drastic, sharp ascents observed under the fed condition (e.g., Fig. 4D, E, F). Visual comparison shows that the GITT model’s predicted concentrations tended to underestimate C_max_, notably evident in cases where the referent exhibited multiple peaks, e.g., analog Subjects 3 and 6 under the fasting condition. In comparison, analogs generally performed well in approaching C_max_ and t_max_, even in cases where the profile exhibited aberrant, complex features. One noteworthy exception was analog Subject 5′s plasma profile under the fed condition (Fig. 4E), which displayed a peculiar, sharp drop in plasma concentration before ascending rapidly to attain C_max_. The analog and GITT model both failed to produce close approximations to C_max_ and t_max_ although the analog result was somewhat closer to the referent C_max_ and sharply lower concentration around 3.25 h. On the other hand, the analog did relatively well in predicting Subject 4′s plasma concentrations (Fig. 3D, 4D) that increased steeply to reach C_max_ that far exceeded the peak concentration in all other profiles (up to twelve-fold differences).

**Table 2:** Subject-specific parameter values used for validation (fasting/fed).

	Subject					
Parameter	1	2	3	4	5	6
*XScale*	0.45	0.45	0.45	0.45	0.45	0.45
*YScale*	6800/6200	6800/6200	6800/6200	600/560	6800/2800	6800/3100
*DtoGDelay*	0/4	1	0	3/4	1/2	0/3
*DtoGFract*	0.6/0.25	0.3/0.7	0.5/0.6	0.7	0.8	0.3/0.5
*DtoGProb*	0.8	0.5/0.8	0.6	0.8	0.9	0.5
*DiffGRatio*	0.15/0.25	1/0.2	0.7/0.05	0.7/0.05	0.6/0.2	0.8/0.3
*GtoCDelay*	0	0	0	0	0	0
*GtoCFract*	0.3	0/0.2	0.2	0.2/0.1	0.2	0.3/0.1
*GtoCProb*	0.2/0.3	0/0.4	0.3/0.2	0.2/0.1	0.2/0.3	0.2
*GAtoPDelay*	0	0	0	0	0	0
*GAtoPFract*	0.9	0.9	0.6	0.32/0.7	0.6	0.6
*GAtoPProb*	0.9	0.9	0.8	0.5	0.5/0.7	0.5
*GBtoPDelay*	6/11	6/5	10/7	7/9	6/9	14/7
*GBtoPFract*	0.3/0.5	0.3/0.8	0.8/0.5	0.6/0.45	0.8/0.75	0.8
*GBtoPProb*	0.3/0.5	0.3/0.7	0.9/0.5	0.5	0.5/0.8	0.5
*GCtoPDelay*	10/9	0/9	8/4	8/9	8/5	8/12
*GCtoPFract*	0.35/0.5	0/0.9	0.9/0.8	0.6	0.8	0.8
*GCtoPProb*	0.3/0.6	0/0.7	0.8/0.7	0.6	0.7	0.8
*PtoEDelay*	0	0	0	0	0	0
*PtoEFract*	0.35/0.2	0.3	0.33/0.3	0.6/0.55	0.35/0.8	0.3/0.4
*PtoEProb*	0.8	0.6/0.35	0.7/0.45	0.8/0.7	0.5/0.6	0.8

A noncompartmental analysis was conducted to estimate pharmacokinetic parameters from the analog’s plasma profiles, and confirm similarity between the in silico and referent pharmacokinetics. The estimated parameters are shown in Table 3. In agreement with our visual assessment, C_max_ and t_max_ estimations were similar between the in silico and referent outcomes. AUC_all_ was also similar although the in silico profiles had somewhat higher estimations. Small differences were noted in the lag time (t_lag_) for the fed condition but not the fasting condition. Peak plasma concentration and lag time as well as AUC_all_ estimations had large variance, partly attributed to Subject 4′s plasma concentration-time measurements that deviated considerably from the other plasma profiles as noted above. The outlier effect was especially evident in the fasting condition where the standard deviation exceeded the mean for all three parameters (Table 3).

**Table 3:** Pharmacokinetic parameter estimates.

Parameter	Observed		In Silico	
	Fasting	Fed	Fasting	Fed
t_lag_ (h)	0.4±0.5*^a^*	1.6±1.2*^a^*	0.4±0.5	1.1±0.7
t_max_ (h)	4.1±0.8	4.5±1.0	4.1±0.8	4.7±0.9
C_max_ (nmol/l)	16.7±21.8	27.3±29.4	16.8±22.3	26.9±28.8
AUC_all_ (h·nmol/l)	53.9±56.9	54.3±47.6	55.3±56.3	57.4±52.1

t_lag_, lag time; t_max_, peak concentration time; C_max_, peak concentration; AUC_all_, area under the plasma concentration-time curve from dosing up to the last time point. Data expressed as mean ± SD. *^a^* From [8].

## Discussion

**Figure 5:**
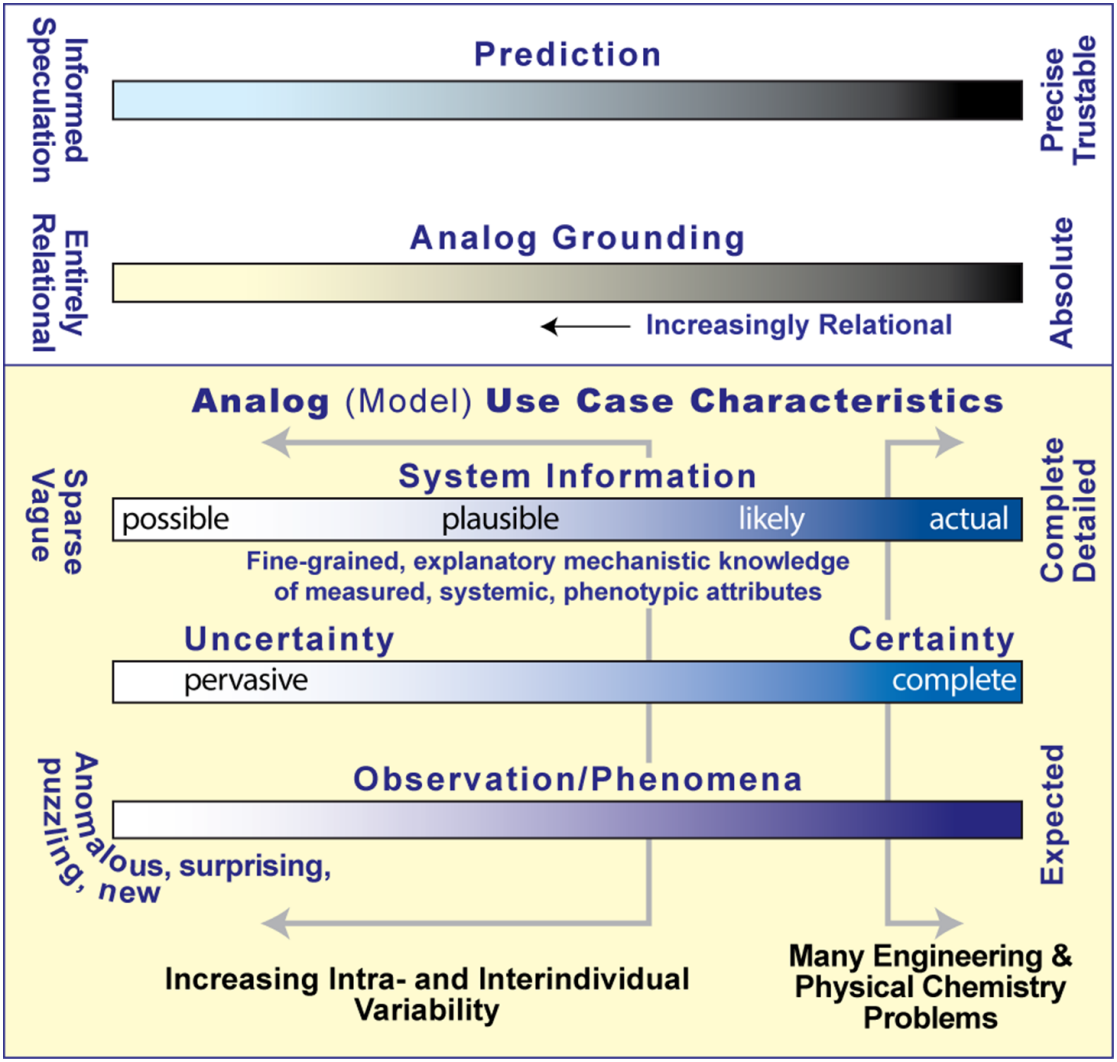
Analog use cases. Each analog experiment (a use case) focuses on one or more aspects of its referent, e.g., felodipine plasma profiles. The particular referent can be characterized by its approximate location on each of the four lower spectra. Those locations guide analog grounding decisions and constrain the characteristics of predictions that the validated analog can make. An analog, in turn, can be located anywhere along a spectrum of software devices (models) ranging from synthetic (used herein) to purely inductive models. Conditions on the far right of the three lower spectra are supportive of continuous mathematical models that are constrained by their formalism and rely on absolute grounding that, upon validation, can make trustable, precise predictions. When center-left, where explanatory, mechanistic knowledge is scarce, the focus needs to shift to mechanism discovery, explanation, and exploration, which are facilitated by relational grounding [7], [31], and reliance on the agent-oriented, discrete event methods used herein is most appropriate.

Modeling and simulation scenarios have been discussed in which conventional pharmacokinetic methods are most useful [4], [5], [7]. They would have well characterized dosage forms, established in vitro-to-in vivo correlation (IVIVC), little intra-individual variability, and explainable interindividual variability supported by ample quantitative data. Such cases can be characterized by locations substantially right-of-center on the Fig. 5 use case spectra, which favors validation of inductive data models that can produce trustable predictions. One’s location shifts left when dealing with complicated pharmacokinetics (e.g., complex, extended-release formulations) because uncertainty increases and precise knowledge diminishes. The generators of underlying phenomena are unclear. The reliable, quantitative data that would be needed to discern and validate (or falsify) plausible generative mechanisms are typically lacking or scarce. When intra- and interindividual variability increases, one’s location shifts further left, and risks of relying on inductive methods and imposed, idealized assumptions increase dramatically. When left-of-center, conventional approaches can fail because one or more assumptions on which the formal analytic approach rests are invalid, or different mechanisms apply to subsets of individuals, but not on all occasions. In those situations, different modeling methods, like those presented herein, are needed to begin developing explanatory mechanistic knowledge that can be exploited to guide formulation design, pharmacokinetic profiling, clinical trial enrichment strategies, etc.

For the extended release felodipine formulation, food interaction is an important factor that alters the drug’s pharmacokinetics, but the underlying mechanisms are mostly unknown [8], a circumstance corresponding to being center-left in Fig. 5. Plasma profiles also exhibit unusual, unexplained, individual variability with or without concurrent food intake. Multiple peaks appear in some but not all individual profiles. When confronted with such complex phenomena, it is reasonable to posit that there can be multiple, different generators causing those unusual plasma profiles. If reality is center-left in Fig. 5, then a seemingly plausible, inductive, conceptual mechanistic hypothesis can be flawed in ways that are not obvious until that mechanism is instantiated (made real, concrete) and executed. See [25] for several examples and a detailed discussion. We argue that it is scientifically prudent to explore multiple mechanistic hypotheses and clearly falsify some of them using in silico experimentation. That is because any complex phenomenon can have multiple, equally valid and plausible generators [33]. It would be particularly useful, given appropriate subject-specific data, to discover one or more mechanistic hypotheses that are equally explanatory for all subjects as demonstrated by achieving prespecified validation targets. A purpose of the synthetic, object- and agent-oriented M&S approach used herein is to both fill out and then shrink the space of plausible mechanisms missed by conventional inductive methods [7]. It provides means–particularly in situations characterized by multisource uncertainty and scarce knowledge–to explore and discover one or more equally explanatory mechanistic hypotheses that can be parameterized to generate individualized plasma profiles that match their counterpart quantitatively. This study demonstrates important, early progress toward those goals.

**Figure 6:**
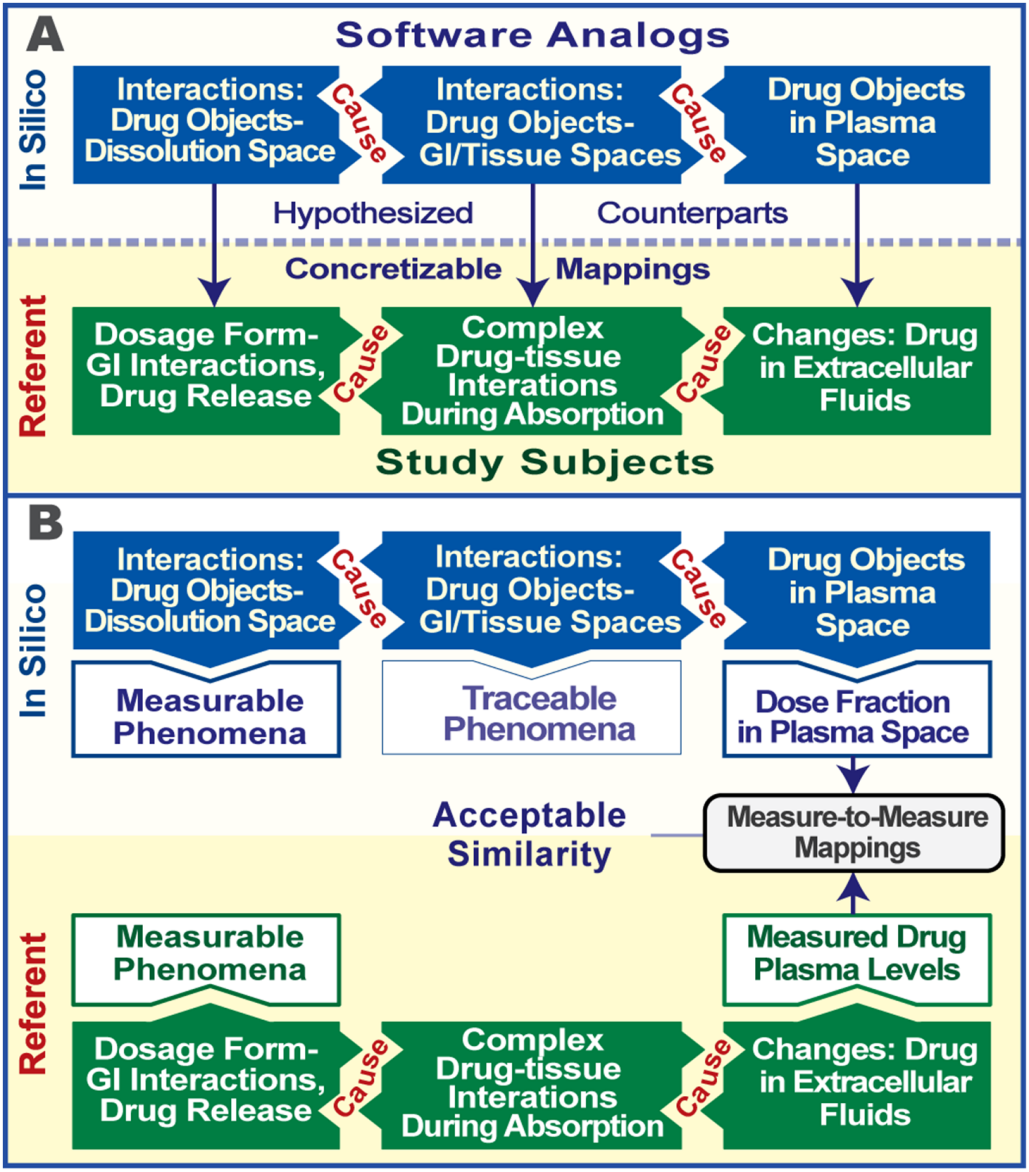
Analog–subject mappings. The engineering objective is to have biomimetic software mechanisms (blue) in which we are building confidence that events occurring during simulations, at different granularities, mimic corresponding events at comparable granularities hypothesized to occur in subjects (green) following dosing. A. Generative mechanisms, phenomena, and their causes occurring during simulations stand as hypotheses (currently quite abstract–coarse grain) about counterparts occurring within each subject. Because all analog events are concrete, those hypotheses are also concretizable mappings. B. The immediate goal is to achieve acceptable, quantitative similarity criteria (measure-to-measure mappings) between each subject’s felodipine plasma profile features and its analog’s counterpart. As similarity criteria are achieved, our confidence increases that simulation details may be predictive. Importantly, influential events within each space can be traced during execution and so can be falsified (or not) by future wet-lab or clinical observations.

Specific, limiting problems in attempting to model fit the felodipine data have been discussed from the classical modeling perspective [17]. Some complications, such as having to reformulate mathematically the inherently discrete, noncontinuous aspects of drug movement and disposition, are easily resolved using the approach described herein. Prediction of individual pharmacokinetics from sparse data is more challenging. The problem is evident with C_max_, which is very important for dosage adjustment and as a measure of exposure to determine maximum therapeutic or adverse effect [34]. The GITT model performs better than an empirical lag-time model [17], however, its predicted profiles tend to underestimate C_max_, which, if consistent, may suggest confounding systematic bias or invalid assumptions. Part of the argument being made for concurrent and synergistic use of synthetic methods is that focusing on discovering and building plausible, generative mechanisms parsimoniously reduces the risk of instantiating such conceptual bias. The validated analog plasma profiles (Figs. 3 and 4) provide evidence supporting that argument. The observed similarity between in silico and clinical plasma profiles establishes a degree of confidence in analog-to-referent mappings (Fig. 6), even though the actual events and processes in the two systems are very different. Having satisfied the plasma profile similarity criteria, the simulations stand as challengeable yet tested theories about events that may have occurred within subjects following dosing.

A significant M&S challenge, which applies to both classical and synthetic modeling, is the problem of overfitting. In equation-based pharmacokinetic modeling the problem is over-parameterization. Physiologically more ‘realistic’ models are susceptible because they require a large number of individual parameter estimates. Sparse data aggravates the problem. The analogous problem in synthetic modeling occurs when we add more detail than is actually needed to achieve the current use cases and validation targets. When building an analog, there is a strong impulse to add mechanistic details (specific regions of the intestine, for example) simply because we have knowledge of those details and evidence that, under some circumstances, they can influence plasma profile shape. As discussed in [5], doing so too early can lead to overly complex and unnecessary analog features. We minimized those problems by adhering to a strong parsimony guideline as specified previously [13], [14]. So doing also helps avoid inscription error, i.e., the logical fallacy of assuming the conclusion and programming in (consciously or otherwise) aspects of the result we expect to see. Whereas classical inductive methods require a sizable set of quantitatively precise, networked assumptions, many beyond validation, the scientific M&S process requires no comparable assumptions: analog specifications and parameterization are made through iterative experiment and mechanism refinement. Upon achieving validation targets, we simply hypothesize that, at comparable levels of explanatory granularity, analog mechanisms have subject specific counterparts as illustrated in Fig. 6A.

The analog’s coarse grain GI/tissue spaces and event mechanisms are an acknowledgement of multi-source uncertainty, where coarse grain mechanisms serve as placeholders for more fine-grained mechanistic details that will challenge (or support) aspects of the hypothesized mechanisms implemented in Fig. 2. Lam et al. [25] showed how analog mechanisms can be made more fine-grained, more biomimetic, and thus more realistic. A reason for taking the next step and marginally increasing detail would be to identify differences between equally plausible mechanisms that could be challenged using a focused wet-lab experiment, like those described by Weitschies et al. [8]. The set of competing, equally plausible, finer grain analogs would all master the original similarity criteria: they would be equally valid and equally explanatory. Being different at some level of mechanistic detail, there will be event differences during execution, which may reveal tenuous hypotheses that need to be revisited, or may bring into focus new hypotheses–secondary sites of food interaction [35], ancillary absorption pathways [36], transitory drug sequestration [37], [38], etc.–that if later validated may help build confidence in analog predictions and bring us closer to unraveling mechanism culprits that confound current analysis. An experiment that measures counterparts in vitro or in vivo will provide evidence that may falsify aspects of some or all of the competing mechanisms. Our expectation is that such tight coupling of in silico and wet-lab experimentation can generate the evidence needed to identify causes of excessive dosage form performance variability, and suggest formulation strategies based on predictive, prospective simulations to improve oral pharmacokinetics and efficacy.

We further envision integrating the conventional pharmacokinetic modeling approach with synthetic methods used herein. Doing so is not straightforward partly because appropriate tools are lacking within the pharmaceutical research and development domain to contemporaneously support and bridge the two approaches. One harmonization strategy is to augment existing pharmacokinetic modeling platforms with object-oriented, discrete event modeling capabilities. So doing may seem somewhat futuristic, but technologies and technical underpinnings for enabling heterogeneous M&S capabilities are in use today in different engineering domains [39]–[41]. In the case of NONMEM or ADAPT, language updates and extensions to Fortran are now available to support OOP [42] as well as discrete event simulations [43]. They subsume older, Fortran-based constructs and enable new object-oriented system components to coexist, execute, inform, and interface with procedural subroutines (e.g., PREDPP) within a common, computational framework. New user functionalities will be feasible, such as rapid virtual prototyping and automated (supervised or unsupervised) model evolution, which can facilitate large-scale, exploratory, mechanism-focused pharmacological M&S.

A key benefit of synthetic, object-oriented M&S is the ease with which existing details can be altered and/or new details added. It is relatively straightforward to “drill down” to explore finer grain mechanism theories while still being parsimonious. For example, objects representing different cell types (e.g., enterocytes) that contain other objects that map to molecules–metabolic enzymes, drug and efflux transporters, etc.–can be added to individual grid locations as done in [13]. The goal might be to explore plausible answers to a question like this: is there a single, somewhat finer grain analog, in which one or more features are common for all subjects, that behaves individually the same as the coarse grain mechanism? The approach is described as tunable resolution [44]. If the answer is yes, we may be getting closer to identifying the problematic drug-absorption-food interaction features, possibly related to BCS class types. So doing would make it easier to explain and enhance product performance. We anticipate that these expanded, exploratory methods will enable solving such long-standing mysteries as why some oral drugs are highly variable beyond what is currently related to BCS classification. Improved, explanatory, mechanistic insight is expected to foster technological innovations to better control variability and improve individual treatment outcomes.

## Conclusion

We used the investigational, in silico framework in Fig. 1 to explore plausible mechanistic hypotheses for the highly variable, complex pharmacokinetics of an extended-release felodipine formulation. The framework comprises an object-oriented, discrete event, whole-body analog supported by features for semi-automated experimentation and analysis. Subject-specific parameterizations enabled each executed analog’s plasma profile to quantitatively mimic features of corresponding individual subject plasma profiles with food interaction. These new methods provide much-needed M&S means to begin unraveling causative mechanisms underlying complex pharmacological phenomena and accelerate progress toward truly personalized medicine.

## Supporting Information

File S1 **Simulated disposition outcomes following changes in analog parameterization.** Starting with the analog parameterized to Table 1′s default values, we explored different parameter values to observe changes in plasma concentration-time profiles. Analog drug concentrations were measured in dose fraction and recorded each time step following the same protocol described in Methods.
(PDF)

## References

[1] Agoram B, Woltosz WS, Bolger MB (2001) Predicting the impact of physiological and biochemical processes on oral drug bioavailability. Adv Drug Deliv Rev 50 Suppl 1: S41–67. 11576695 10.1016/s0169-409x(01)00179-x

[2] Zhou H (2003) Pharmacokinetic strategies in deciphering atypical drug absorption profiles. J Clin Pharmacol 43: 211–227. 12638389 10.1177/0091270002250613

[3] Godfrey KR, Arundel PA, Dong Z, Bryant R (2011) Modelling the double peak phenomenon in pharmacokinetics. Comput Methods Programs Biomed 104: 62–69. 20381191 10.1016/j.cmpb.2010.03.007

[4] Hunt CA, Kennedy R, Kim SHJ, Ropella G (2013) Agent-based modeling: a systematic assessment of uses and requirements for enhancing pharmaceutical research and development productivity. Wiley Interdiscip Rev Syst Biol Med 5: 461–480. 23737142 10.1002/wsbm.1222 PMC3739932

[5] Kim SH, Jackson AJ, Hur R, Hunt CA (2012) Individualized, discrete event, simulations provide insight into inter- and intra-subject variability of extended-release, drug products. Theor Biol Med Model 9: 39. 22938185 10.1186/1742-4682-9-39 PMC3563477

[6] Fisher J, Henzinger TA (2007) Executable cell biology. Nat Biotechnol 25: 1239–1249. 17989686 10.1038/nbt1356

[7] Hunt CA, Ropella GE, Lam TN, Tang J, Kim SH, et al (2009) At the biological modeling and simulation frontier. Pharm Res 26: 2369–2400. 19756975 10.1007/s11095-009-9958-3 PMC2763179

[8] Weitschies W, Wedemeyer RS, Kosch O, Fach K, Nagel S, et al (2005) Impact of the intragastric location of extended release tablets on food interactions. J Control Release 108: 375–385. 16213057 10.1016/j.jconrel.2005.08.018

[9] Löbenberg R, Amidon GL (2000) Modern bioavailability, bioequivalence and biopharmaceutics classification system. New scientific approaches to international regulatory standards. Eur J Pharm Biopharm 50: 3–12. 10840189 10.1016/s0939-6411(00)00091-6

[10] Yasir M, Asif M, Kumar A, Aggarval A (2010) Biopharmaceutical classification system: an account. Int J PharmTech Res 2: 1681–1690.

[11] Kim EJ, Chun MK, Jang JS, Lee IH, Lee KR, et al (2006) Preparation of a solid dispersion of felodipine using a solvent wetting method. Eur J Pharm Biopharm 64: 200–205. 16750355 10.1016/j.ejpb.2006.04.001

[12] Bazzo GC, Caetano DB, Boch ML, Mosca M, Branco LC, et al (2012) Enhancement of felodipine dissolution rate through its incorporation into Eudragit E-PHB polymeric microparticles: in vitro characterization and investigation of absorption in rats. J Pharm Sci 101: 1518–1523. 22228026 10.1002/jps.23044

[13] Tang J, Hunt CA (2010) Identifying the rules of engagement enabling leukocyte rolling, activation, and adhesion. PLoS Comput Biol 6: e1000681. 20174606 10.1371/journal.pcbi.1000681 PMC2824748

[14] Engelberg JA, Datta A, Mostov KE, Hunt CA (2011) MDCK cystogenesis driven by cell stabilization within computational analogues. PLoS Comput Biol 7: e1002030. 21490722 10.1371/journal.pcbi.1002030 PMC3072361

[15] Bergstrand M, Söderlind E, Weitschies W, Karlsson MO (2009) Mechanistic modeling of a magnetic marker monitoring study linking gastrointestinal tablet transit, in vivo drug release, and pharmacokinetics. Clin Pharmacol Ther 86: 77–83. 19387437 10.1038/clpt.2009.43

[16] Bergstrand M, Söderlind E, Eriksson UG, Weitschies W, Karlsson MO (2012) A semi-mechanistic modeling strategy to link in vitro and in vivo drug release for modified release formulations. Pharm Res 29: 695–706. 21948457 10.1007/s11095-011-0594-3

[17] Hénin E, Bergstrand M, Standing JF, Karlsson MO (2012) A mechanism-based approach for absorption modeling: the Gastro-Intestinal Transit Time (GITT) model. AAPS J 14: 155–163. 22286919 10.1208/s12248-012-9324-y PMC3326175

[18] Weitschies W, Kötitz R, Cordini D, Trahms L (1997) High-resolution monitoring of the gastrointestinal transit of a magnetically marked capsule. J Pharm Sci 86: 1218–1222. 9383729 10.1021/js970185g

[19] Bonabeau E (2002) Agent-based modeling: methods and techniques for simulating human systems. Proc Natl Acad Sci U S A 99 Suppl 3: 7280–7287. 12011407 10.1073/pnas.082080899 PMC128598

[20] Grimm V, Railsback SF (2005) Individual-based modeling and ecology. Princeton: Princeton University Press. 480 p.

[21] Macal CM, North MJ (2010) Tutorial on agent-based modeling and simulation. J Simul 4: 151–162.

[22] Amigoni F, Schiaffonati V (2007) Multiagent-based simulation in biology: a critical analysis. In: Magnani L, Li P, editors. Model-based reasoning in science, technology, and medicine. Berlin: Springer-Verlag. pp. 179–191.

[23] An G, Mi Q, Dutta-Moscato J, Vodovotz Y (2009) Agent-based models in translational systems biology. Wiley Interdiscip Rev Syst Biol Med 1: 159–171. 20835989 10.1002/wsbm.45 PMC3640333

[24] Wirfs-Brock R, Wilkerson B, Wiener L (1990) Designing object-oriented software. Upper Saddle River: Prentice Hall. 341 p.

[25] Lam TN, Hunt CA (2009) Discovering plausible mechanistic details of hepatic drug interactions. Drug Metab Dispos 37: 237–246. 18936110 10.1124/dmd.108.023820

[26] Park S, Kim SH, Ropella GEP, Roberts MS, Hunt CA (2010) Tracing multiscale mechanisms of drug disposition in normal and diseased livers. J Pharmacol Exp Ther 334: 124–136. 20406856 10.1124/jpet.110.168526

[27] Zeigler BP, Praehofer H, Kim TG (2000) Theory of modeling and simulation: integrating discrete event and continuous complex dynamic systems. San Diego: Academic Press Professional. 510 p.

[28] Robertson DA (2005) Agent-based models to manage the complex. In: Richardson KA, editor. Managing organizational complexity: philosophy, theory, and application. Charlotte: Information Age Publishing. pp. 417–430.

[29] Epstein B (2012) Agent-based modeling and the fallacies of individualism. In: Humphreys P, Imbert C, editors. Models, simulations, and representations. New York: Routledge. pp. 115–144.

[30] Luke S, Cioffi-Revilla C, Panait L, Sullivan K, Balan G (2005) MASON: a multi-agent simulation environment. Simulation 82: 517–527.

[31] Hunt CA, Ropella GEP, Lam TN, Gewitz AD (2011) Relational grounding facilitates development of scientifically useful multiscale models. Theor Biol Med Model 8: 35. 21951817 10.1186/1742-4682-8-35 PMC3200146

[32] Schildt H (2011) Java: the complete reference, 8th edition. New York: McGraw-Hill. 1152 p.

[33] Ingo B, Alan L (2012) Reductionism in biology. The Stanford Encyclopedia of Philosophy. Available: http://plato.stanford.edu/archives/sum2012/entries/reduction-biology Accessed 2014 July 15.

[34] Food and Drug Administration (2003) Exposure-response relationships – study design, data analysis, and regulatory applications. Guidance for Industry. Available: http://www.fda.gov/downloads/Drugs/GuidanceComplianceRegulatoryInformation/Guidances/UCM072109.pdf Accessed 2014 July 15.

[35] Trevaskis NL, Charman WN, Porter CJ (2008) Lipid-based delivery systems and intestinal lymphatic drug transport: a mechanistic update. Adv Drug Deliv Rev 60: 702–716. 18155316 10.1016/j.addr.2007.09.007 PMC7103284

[36] Hussain N, Jaitley V, Florence AT (2001) Recent advances in the understanding of uptake of microparticulates across the gastrointestinal lymphatics. Adv Drug Deliv Rev 50: 107–142. 11489336 10.1016/s0169-409x(01)00152-1

[37] Mrsny RJ (1992) The colon as a site for drug delivery. J Control Release 22: 15–34.

[38] Abrahamsson B, Alpsten M, Hugosson M, Jonsson UE, Sundgren M, et al (1993) Absorption, gastrointestinal transit, and tablet erosion of felodipine extended-release (ER) tablets. Pharm Res 10: 709–714. 8321836 10.1023/a:1018959732744

[39] MacMillen D, Camposano R, Hill D, Williams TW (2000) An industrial view of electronic design automation. IEEE Trans Comput Aided Des Integr Circuits Syst 19: 1428–1448.

[40] Travis J, Kring J (2006) LabVIEW for everyone: graphical programming made easy and fun. Upper Saddle River: Prentice Hall. 1032 p.

[41] Eker J, Janneck JW, Lee EA, Liu J, Liu X, et al (2003) Taming heterogeneity – the Ptolemy approach. Proc IEEE 91: 127–144.

[42] Clerman NS, Spector W (2011) Modern Fortran: style and usage. New York: Cambridge University Press. 352 p.

[43] Pooch UW, Wall JA (1992) Discrete event simulation: a practical approach. Boca Raton: CRC Press. 432 p.

[44] Kirschner DE, Hunt CA, Marino S, Fallahi-Sichani M, Linderman JJ (2014) Tuneable resolution as a systems biology approach for multi-scale, multi-compartment computational models. Wiley Interdiscip Rev Syst Biol Med 6: 289–309. 24810243 10.1002/wsbm.1270 PMC4102180

