# Supplementary material for: In Silico, Experimental, Mechanistic Model for Extended-Release Felodipine Disposition Exhibiting Complex Absorption and a Highly Variable Food Interaction
Source: PLoS One. 2014 Sep 30;9(9):e108392. doi: 10.1371/journal.pone.0108392 (PMC4182452; doi:10.1371/journal.pone.0108392)
Supplement: File S1 — Simulated disposition outcomes following changes in analog parameterization. Starting with the analog parameterized to Table 1′s default values, we explored different parameter values to observe changes in plasma concentration-time profiles. Analog drug concentrations were measured in dose fraction and recorded each time step following the same protocol described in Methods. (PDF) [file pone.0108392.s001.pdf]

SUPPORTING INFORMATION

In Silico, Experimental, Mechanistic Model for Extended-Release Felodipine Disposition Exhibiting Complex Absorption and a Highly Variable Food Interaction

Sean H. J. Kim, Andre J. Jackson, C. Anthony Hunt

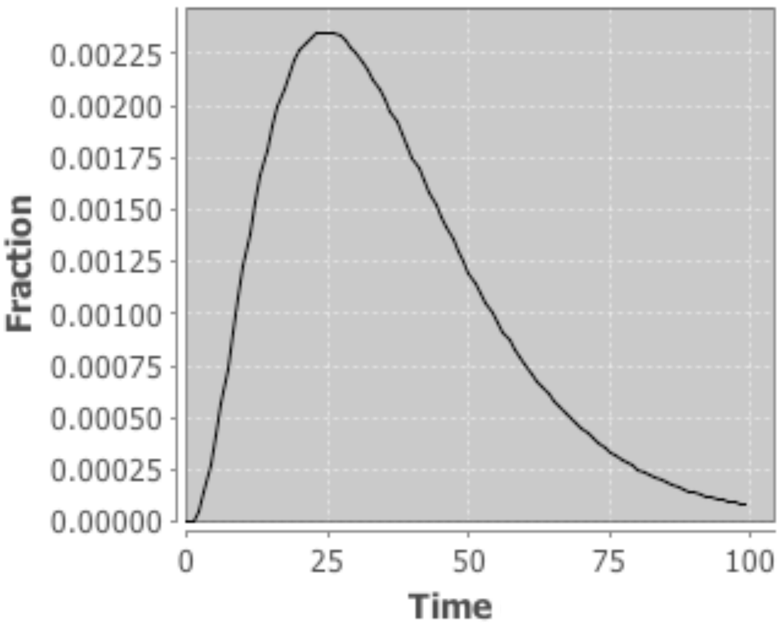

Figure S1. Plasma concentration-time profile of an analog parameterized to Table 1's default values.

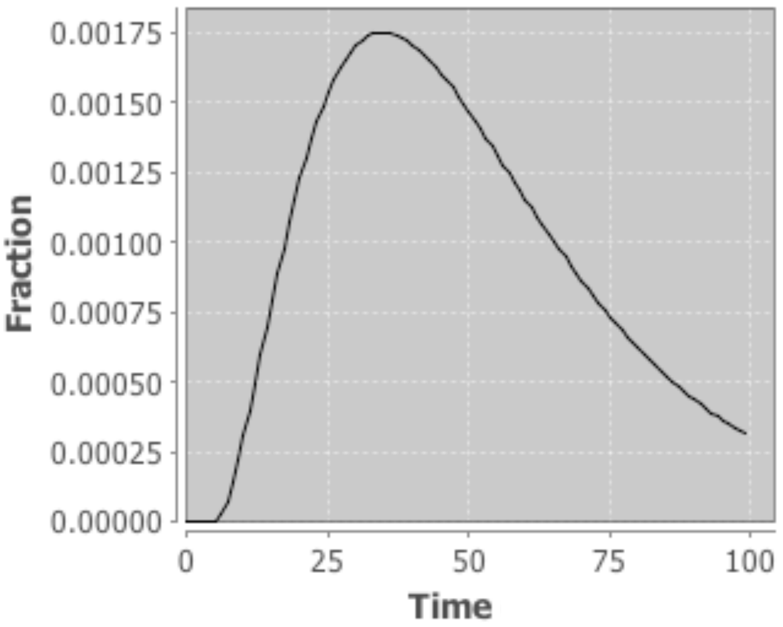

Figure S2. Plasma concentration-time profile of the analog from Fig. S1 with *DtoGDelay* = 5 (default = 1) and *DtoGProb* = 0.4 (default = 0.8).

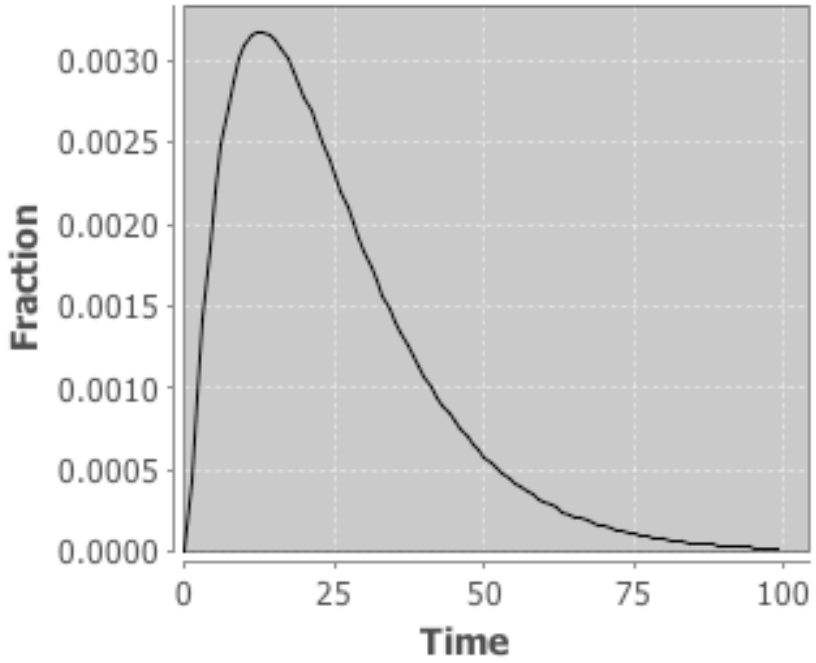

Figure S3. Plasma concentration-time profile of the analog from Fig. S1 with *DtoGFract* set to 0.8 (default = 0.1).

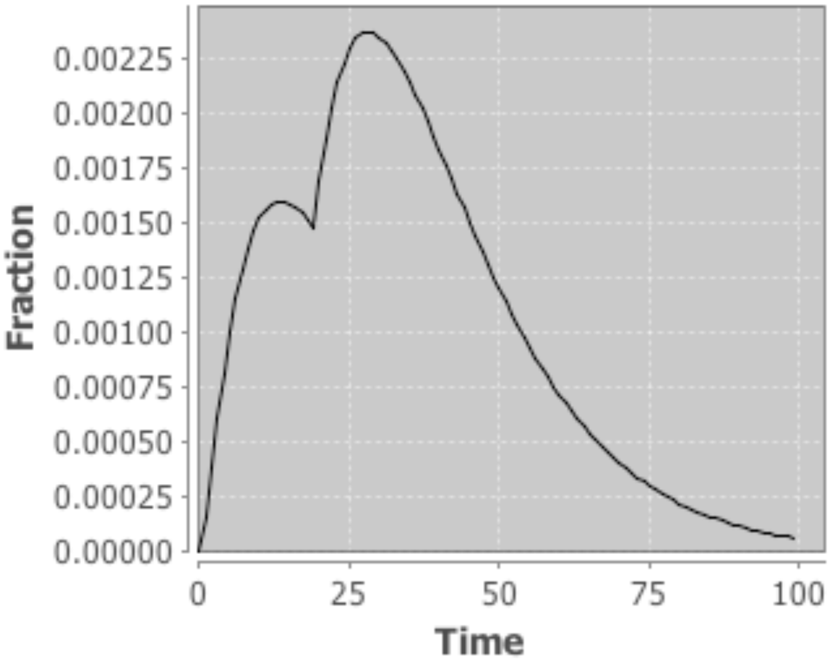

Figure S4. Plasma concentration-time profile of the analog from Fig. S1 with *DiffGRatio* = 0.5 (default = 1) and *GAtoPFract* = 0.6 (default = 0.1).

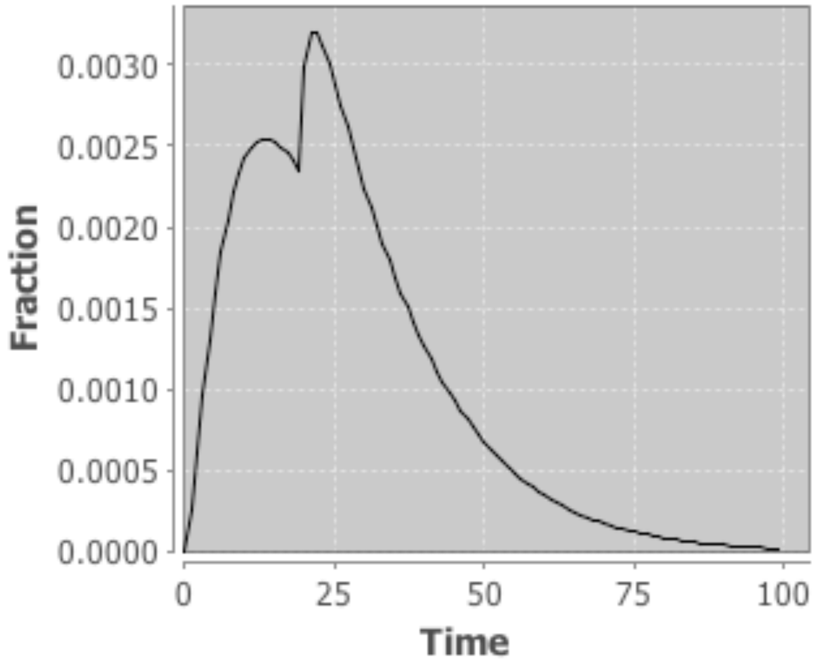

Figure S5. Plasma concentration-time profile of the analog from Fig. S1 with *DiffGRatio* = 0.8 (default = 1), *GAtoPFract* = 0.6 (default = 0.1), and *GBtoPFract* = 0.7 (default = 0.1).

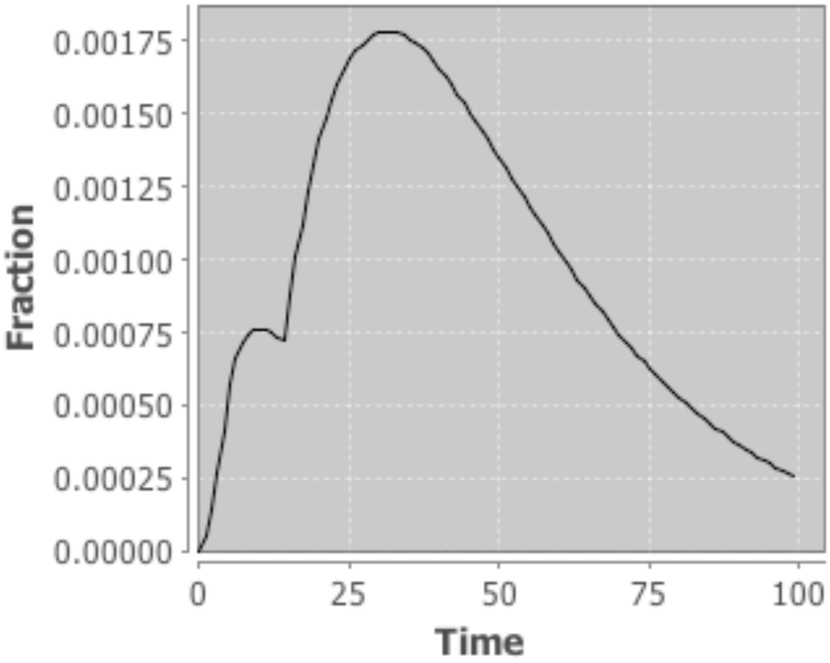

Figure S6. Plasma concentration-time profile of the analog from Fig. S1 with *GtoCDelay* = 5, *GtoCFract* = 0.6, *GtoCProb* = 0.7, *GCtoPDelay* = 15, *GCtoPFract* = 0.2, and *GCtoPProb* = 0.2, which specify drug movement to and from GI/tissue space C.

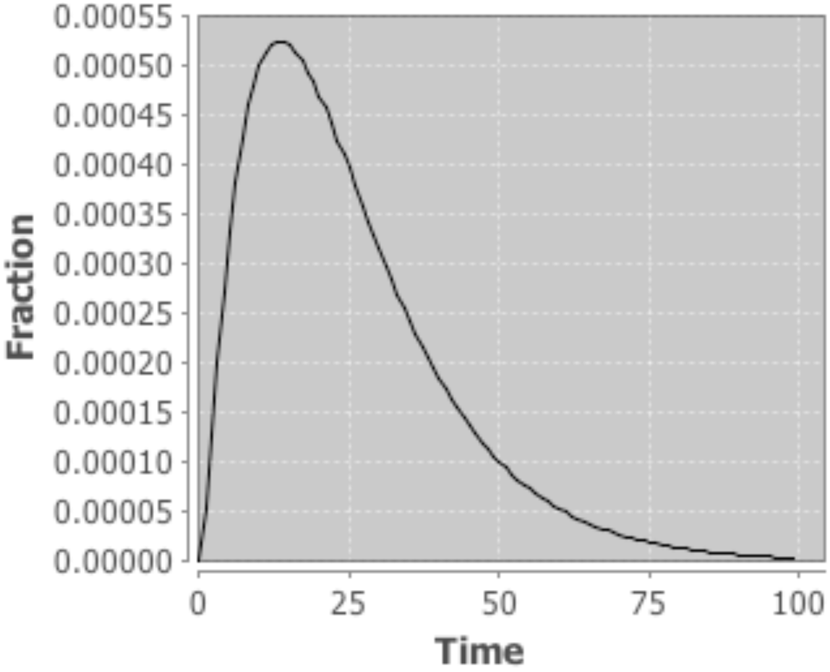

Figure S7. Plasma concentration-time profile of the analog from Fig. S1 with *PtoEFract* set to 0.6 (default = 0.1).

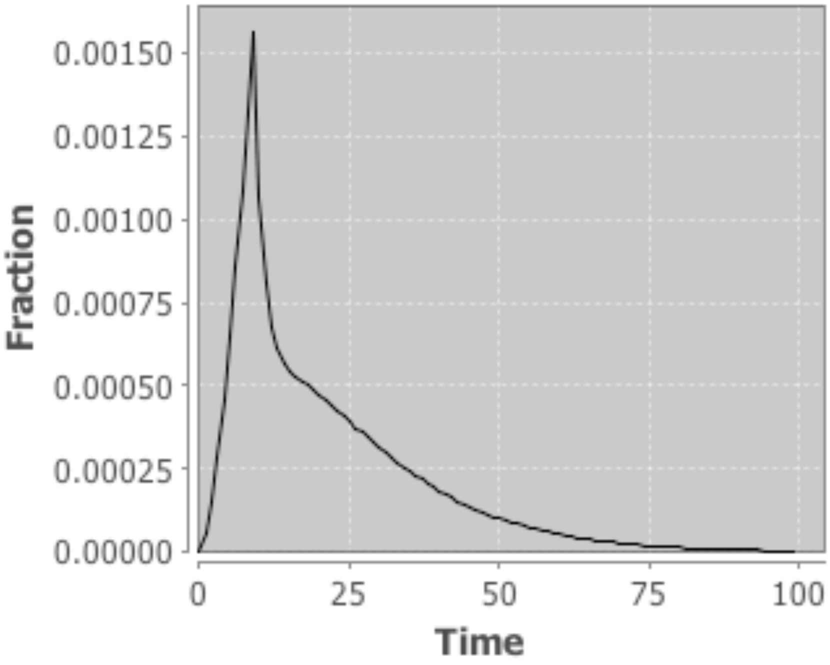

Figure S8. Plasma concentration-time profile of the analog from Fig. S1 with *PtoEDelay* = 10 (default = 0) and *PtoEFract* = 0.6 (default = 0.1).

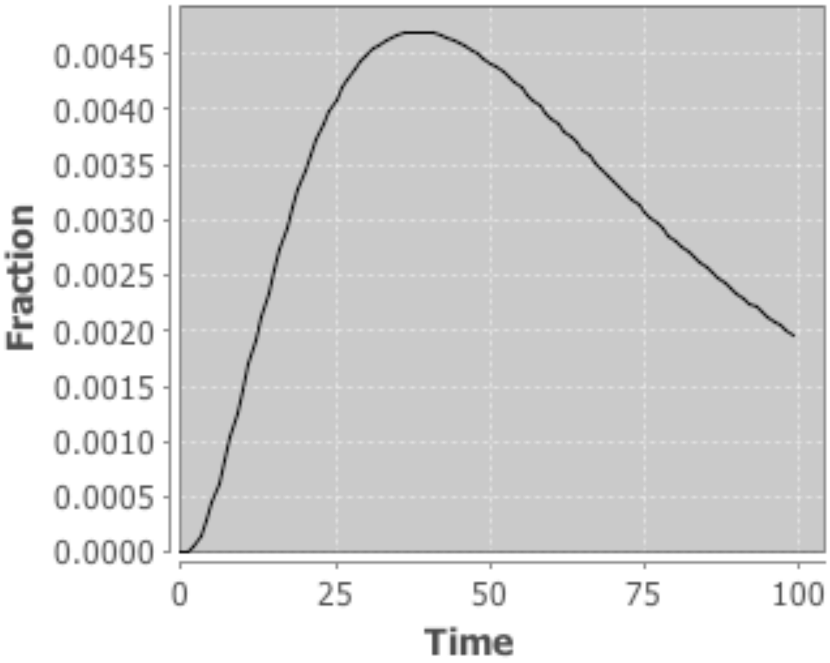

Figure S9. Plasma concentration-time profile of the analog from Fig. S1 with *PtoEProb* set to 0.2 (default = 0.8).

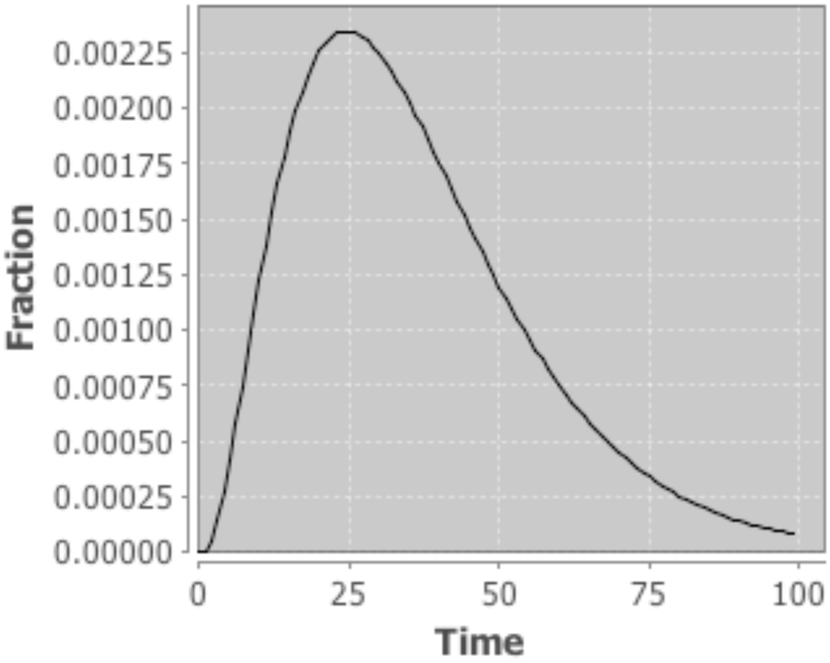

Figure S10. Plasma concentration-time profile of the analog from Fig. S1 with *InitDose* increased to 50000 (default = 10000). No change in plasma profile is expected, which is measured in dose fraction.
